# Solid Sorbents as a Retrofit Technology for CO_2_ Removal from Natural Gas Under High Pressure and Temperature Conditions

**DOI:** 10.1038/s41598-019-57151-x

**Published:** 2020-01-14

**Authors:** Majeda Khraisheh, Fares Almomani, Gavin Walker

**Affiliations:** 1 Qatar University, Department of Chemical Engineering, College of Engineering, P.O. Box 2713, Doha, Qatar; 2 Department of Chemical Sciences, SSPC, Bernal Institute, University of Limerick, Limerick, Republic of Ireland

**Keywords:** Carbon capture and storage, Nanoscale materials

## Abstract

The capture of CO_2_ under high pressure and temperature is challenging and is required in a number for industrial applications including natural gas processing. In this work, we examine the use of benchmark hybrid ultraporous materials HUMs for their potential use in CO_2_ adsorption processes under high-pressure conditions, with three varying temperatures (283, 298 and 318 K). NbOFFOVE-1-Ni and SIFSIX-3-Ni were the selected HUMs given their established superior CO_2_ capacity under low pressure (0–1 bar). Both are microporous with highly ordered crystalline structures as compared to the mesoporous hexagonal silica (Santa Barbara Anhydrous-15 (SBA-15)). SBA-15 was previously tested for both low and high-pressure applications and can serve as a benchmark in this study. Sorbent characterization using XRD, SEM, FTIR and N_2_ adsorption were conducted to assure the purity and structure of the sorbents. TGA analysis were conducted to establish the thermal stability of the sorbents under various temperatures. High-pressure CO_2_ adsorption was conducted from 0–35 bar using magnetic suspension balance (Rubotherm). Although the SBA-15 had the highest surface (527 m^3^/g) are of the three adsorbents, the CO_2_ adsorption capacity (0.42 mmol/g) was an order of magnitude less than the studies HUMs with SIFSIX-3-Ni having 2.6 mmol/g, NbOFFIVE-1-Ni achieving 2.5 mmol/g at 298 K. Multistage adsorption isotherms were obtained at different pressures. In addition, results indicate that electrostatics in HUMs are most effective at improving isosteric heat of adsorption *Q*_st_ and CO_2_ uptake. Higher temperatures had negative effect on adsorption capacity for the HUMs and SBA-15 at pressures between 7–9 bar. In SAB-15 the effect of temperature is reversed in what is known as a cross over phenomena.

## Introduction

Carbon dioxide (CO_2_) emissions and its association with green gashouses and fossil fuels have been the focus of much attention in the media, scientific and public communities^1^. The worldwide demand for energy is continuously rising and although great efforts are placed on finding alternative energy resources, more than 85% of worldwide energy consumption is fossil fuel related^2,3^. Around 7 thousand million metric tons (MMT) of greenhouse gases (GHG) are approximately discharged into the atmosphere.

Leading world economies, such as China, use coal fired power plants to satisfy its ever-growing energy and industrial demands as mandated by the abundance of its coal reserves and its low cost^4^. Around 80% of the country’s power is associated with burning coal, while neighboring India produces two thirds of its electricity using coal. Many other leading western economies also rely on coal for power generation. Fossil fuels, in general, and coal in specific are associated with the emission of large volumes of carbon dioxide. CO_2_ is considered a key contributor to climate change as recently reported in the IPCC, 2018 report^5^. Although many technologies exist, carbon capture and storage (CCS) is still one of the most applied technologies used for the mitigation of CO_2_ emissions^2^. The process involves the separating of CO_2_ from its anthropogenic point sources (as in thermal power plants) before compressing, transporting and storing CO_2_ in appropriate underground formation. The capture can be granted post-combustion, pre-combustion and oxyfuel combustion. CO_2_ capture from post combustion can be used in both new and existing CO_2_ emitting industries and power plants and hence has been the focus of many research and technological advancements. Such technologies can be categorized as chemical or physical in nature and are based on adsorption, cryogenic processes and membrane separations^6,7^. Amine scrubbing is one of the most advanced benchmark CO_2_ adsorption process that employs different types of amines^8^.

Pre-combustion is typically applied for conditions where synthesis gas (syngas) is produced at high pressure and high CO_2_ content^1^. The separation of the CO_2_ from such stream is a better obtained, from a thermodynamic and energy intensity standpoint, compared to post combustion treatment. As such applications deals with high temperature and high pressure streams, identifying materials capable of achieving high separation under extreme conditions is highly required^9,10^.

Solid sorbent technology offered a viable CO_2_ capture alternative to overcome some of the energy and regeneration requirements associated with traditional amine based CO_2_ absorption^11^. Solid adsorbents were reported to have lower regeneration energy requirements, better adsorption capacity selectivity^12,13^. More recently, zeolites, ionic liquids, metal organic frame works (MOFs) also known as porous dination polymers^14^, covalent organic frame works^15^ and other materials have been reported with varying degrees of success for CO_2_ capturing and removal under different operating conditions. The use of solid adsorbents has been motivated by their reduced costs of regeneration and activation and can be judged based on the material selectivity, stability, costs and ease of regeneration. In addition, controlling the pore sizing and the functionalization of the adsorbent have been reported as two of the most prominent factors affecting gas separation and uptake^16^. Since post combustion requires the separation to occur at higher pressures and elevated temperature, it is essential to find solid adsorbents that can capture the CO_2_ under such extreme circumstances under many cycles of operation^7,17^.

Belmabkhout^12^ reviewed a number of physical adsorbents for the removal of CO_2_ and the impact of variables on the trace CO_2_ removal. The authors highlighted that the adsorption energies are important factor for the selection of a potential mesoporous solid material in CO_2_ adsorption. High selectivity is vital in mixed gas applications in addition to solid thermal and mechanical stability, and cost effectiveness^12^. From various adsorbents HUMs has been greatly studied recently^18^. Variety of HUMs are available for many different applications. As for CO_2_ adsorption, HUMs can be categorized based on their open vs closed metal frame works^13,19^, narrow pore size HUMs with tailored functional groups that can be achieved pre or post synthesis of the metal frame work. In principle, advanced physisorbents capable of CO_2_ removal from multicomponent gas streams have the potential of offering a new class of materials that can have a great positive impact on the CO_2_ capture technology.

An example of open-ended mesoporous material with interconnected channels is SBA-15^3,20^. The interconnections through the narrow microspores and the reported oxygen vacancies were pivotal in the CO_2_ adsorption under high pressure operations^3^. Zeolites (e.g. zeolite 13X) and metal-organic frameworks (e.g. Mg-HUM-74) form the current standard in the capacity for CO_2_ capture^21^. Some of their main drawbacks are poor selectivity for CO_2_ in gas mixtures, high cost, modest thermal and mechanical stability under high pressure applications. It is evident that a new generation of physisorbents are urgently needed to address the carbon capture applications. Hybrid ultramicroscopic materials have emerged thanks to a crystal engineering approach that enabled systematic study of a previously underexplored class of physisorbents^21,22^. Moreover, they can be synthesized from very cheap starting organic ligands (e.g. pyridine) and metal salts with modular nature SIFSIX-3-M′ (SIFSIX = SiF_6_^−2^, 3 = pyrazine, M′ = Ni, Co, Zn, Cu) is a breed of hybrid ultramicroscopic materials that offers excellent removal of small molecule impurities such as CO_2_. This is attributed to its ability to incorporates strong electrostatics and has ultramicropores that are fine tuned for adsorption of molecules like CO_2_^22^. SIFSIX-3-Ni has been reported to have excellent CO_2_ removal, stability up to 80% relative humidity and in the presence of other sour gases such as H_2_S. All such activity has been investigated at trace CO_2_ concentrations (400 ppm) and low pressures not exceeding 1 bar. NbOFFIVE-1-Ni has also been reported to have superior CO_2_ uptakes 27.6 cm^3^g^−1^ at low CO_2_ concentration and system pressures not exceeding 3 mbar^22^.

Searching through the large volume of literature in the removal of CO_2_ from various gas streams, one can conclude that studies related to high-pressure high temperature operations are scarce^2,3^. In IGCC for example, operating under high pressure and temperature is required to eliminate the need to cool the stream down or adjust the pressure adding to the complexity and cost of the process. Ullah^3^ reported on the adsorption behavior of SBA-15, a mesoporous silica, for pressures reaching 200 bar and three various temperatures while more recently Reiser^23^ reported on the influence of high temperature and pressure (25 MPa) at two temperatures for SBA-15. To the best of our knowledge, reports of high pressure or elevated temperature for SIFSIX-3-Ni and NbOFFIVE-1-Ni are not reported and are considered here as they are known to exhibit benchmark selectivity for CO_2_. Studies on low pressures up to 1 bar and reduced pressure conditions are available in literature. Here, we focus our attention to the study of three sorbents for CO_2_ removal under high-pressure conditions (up to 200 bar) at various temperatures namely NbOFFIVE-1-Ni, SIF SIX-3-Ni. Additionally, we test the SBA-15 as a reference material as literature high temperature studies are available and can be used as a benchmark. The materials were selected due to their strong thermal and mechanical stability, elevated CO_2_ adsorption capacity and ease of synthesis. Rubotherm magnetic suspension balance measurements were conducted at high pressure (35 bar) but can go up to 200 bar, and three different temperatures. Thermal stability tests were conducted under varied temperatures in addition to establishing the adsorption-desorption isotherms for the three materials. Finally, the isosteric heat of adsorption were established for the adsorbents.

## Materials and Methods

All materials and reagents used where of high purity, supplied by Sigma-Aldrich, and used without modifications. Gases were also of high purity and research grade supplied by Buzwair Inc. Qatar.

### Sorbent synthesis

NbOFFIVE-1-Ni is a fluorinated porous HUM and is typically derived from the SIFSIX family by ridging Ni(II)- pyrazine square grid layers with the (NbOF_5_)^−2^ pillars^12,24^. In short, adequate amounts of hydrofluoric acid, pyrazine, niobium (V) oxide and nickel hexahydrate were mixed with 3 ml of deionized water in a 40 ml Teflon bomb. The mixture is heated gradually to 135 °C for 24 hours. The material is allowed to cool for further 24 hours after which the violet colored material is obtained. The material is then rinsed using methanol to ensure no unreacted material is present such as unreacted hydrofluoric acid. The clean sample was then degassed by heating and evacuation for 24 hours at a 10 deg C rate/min to a temperature of 110 °C using dynamic vacuum (<5 μm Hg) devise (SmartVacPrep). Solvothermal reaction at 85 °C method was used to prepare the SIFSIX-3-Ni samples. 1 mmol of Nickel silicofluoride (NiSiF6) and 2 mmol of pyrazine were mixed with 20 ml methanol in a teflon bomb for three days until the resultant bluish powder is formed. As is the case with NbOFFIVE-1-Ni, the powder was washed with methanol to remove any access unreacted war materials. Samples were activated by degassing as described above for 15 hours and a max temperature of evacuation at 75 °C. SBA-15 was prepared in line with reported procedures^3^ with the variation of heating using the microwave oven to heat the slurry to the required temperature. 10 g of pluronic P123 amphiphilic triblock copolymer was mixed with 75 ml of 2 M hydrochloric acid and around 60 ml deionized water. The mixture was heated to around 40 °C while adding slowly 21 g of tetraethyl orthosillicate (TEOS) which is the main organo-silica source. The slurry was mixed well using magnetic stirrer them moved to a microwave and heated for 10 minutes at low power for temperature to reach 85 °C. The sample was washed repeatedly with deionized water and then treated again hydrothermaly for 24 hours at 50 °C. Samples were stored in airtight glass vials for further use and analysis.

### Sample characterization

Thermal stabilities of all samples were conducted using a thermogravimetric analysis (**TGA** analyzer, Perkin Elmer Pyris 6). Samples of about 0.1 g were heated from ambient temperatures to 700 °C at a rate of 10 deg C/min heating under N_2_ atmosphere^25^. Thermograms are recorded for each of the sorbents.

N_2_ gas adsorption was performed at 77 K using liquid nitrogen. All samples were degassed for a minimum of 3 hours prior to characterization at 150 °C under vacuum. The adsorption-desorption analysis were conducted for the three samples under investigation. The Brunauer-Emmett-Teller (BET) method was used to conduct the microporosimetric characterization of the samples using ASAP 2420 surface and porosity analyzer (Micromeritics, Germany). The BET model was used to analyze the surface area in relation to the relative pressure of the system (P/P_o_). Pore size distribution were obtained by the Barrett-Joyner-Halenda (BHJ) method^25,26^.

Powder X-ray diffraction (XRD). Diffractograms were recorded on a Bruker D2 Phaser with range between 2°–75° over 2 Theta (2θ). Furthermore, Fourier transformation infrared spectra (FTIR) (using Bruker Vertex 80) for the three sorbents were conducted in the range 4000–400 cm^−1^.

### High-pressure CO_2_ adsorption experiments

**Figure 1:**
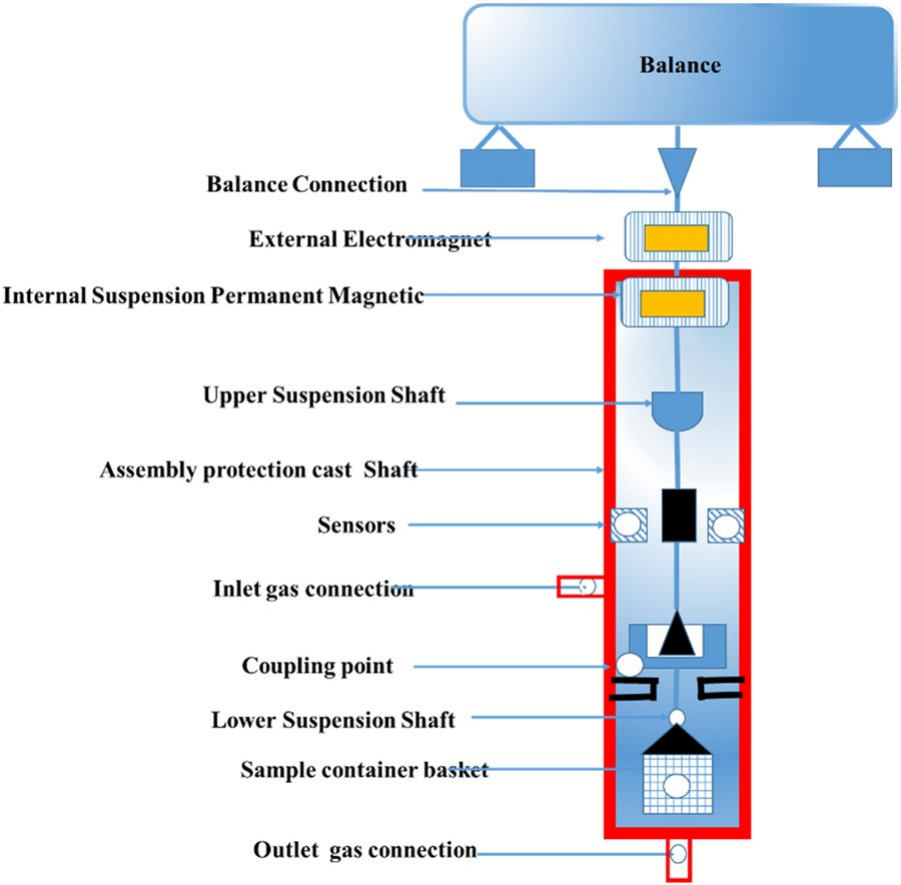
Schematic of High-pressure magnetic suspension (Rubotherm).

In order to study the CO_2_ adsorption and uptake under high-pressure conditions (200 bar, 20 MPa), magnetic suspension balance (MSB) and gas dosing system (GDS) from Rubotherm Prazisionsmesstechnik (Germany) were used. GDS was supported by automated Teledyne Isco 260D pump to pressurize and depressurize (adsorption-desorption) the system in a stepwise fashion. A schematic diagram of the experimental set up is shown in Fig. 1. This apparatus has ability to raise the temperature and pressure up to 350 bar, and 100 °C respectively. The pressure transducers of Paroscientific, USA, were installed to measure the pressure from vacuum to 250 bar with an accuracy of 0.01% bar, and temperature sensor from Minco PRT, USA an accuracy of ± 0.5 °C. Firstly, buoyancy measurements conducted using helium under pressure of around 50 bars. Typically, samples with size between 0.10–0.20 g are placed in the MSB sample holder after degassing. This *in situ* degassing of the samples at 75 °C took place until equilibrium (reached dafter nearly 4 hours) was reached and no weight variation in the system were detected. The measuring chamber was adjusted to the required experimental temperature (283 K, 298 K, 318 K). For each pressure points, equilibrium time of around 80 min was required at a given temperature. The system was fully automated and is programmed to have a step pressure function to ensure regular pressure increase and decrease as required to obtain the adsorption data.

## Results and Discussion

**Figure 2:**
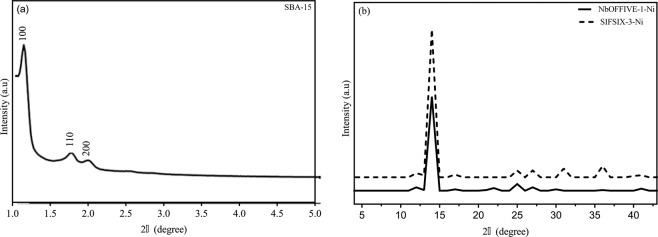
XRD for (**a**) SBA-15 and (**b**) NbOFFIVE-1-Ni and SIFSIX-3-Ni.

The prepared materials were tested to ascertain their bulk purity, identities and crystalline structure using PXRD of a set wavelength. A scattering pattern results when a microcrystalline sample is hit by X-Rays, which can be used to characterize the ordering of the crystalline structure. Figure 2 shows the experimental XRD patterns for the three prepared materials. It can be seen that in all prepared samples, XRD were consistent with the reported crystal structure of the suggesting that the required phase purity was achieved during the experimental preparation with the required synthesized framework. Although deviation from the reported procedure for the preparation of SBA-15 was employed utilizing microwave heating of the samples, the resultant XRD consisted of a typical peaks of high intensity at the 100, 200 and 215 diffraction peaks showing good crystalline structure and consistency of reported data. The intense peak was located around 2θ = 0.90–0.97 followed by two lower intensity peaks at 2θ = 1.51–1.62 and 1.76–1.88. Accordingly, it can be inferred that the use of the microwave treatment is time and cost saving in the case of this particular material preparation. The impact of microwave treatment is a focus of other research studies by the authors and will not be addressed in the context of this manuscript. The resultant XRD patterns for the SBA-15 material after CO_2_ sorption and high-pressure operation still showed the characteristic hexagonal structure peaks of mesoporous silica SBA-15 crystalline structure but indicated that a degree of amorphous character has been induced upon the high-pressure operations but no major suppressing of the crystalline character of the parent material. Weng^27^ reported that the SBA-15 shape took on many hexagonal pillar-liked regions of relatively uniform crystal.

### SEM

**Figure 3:**
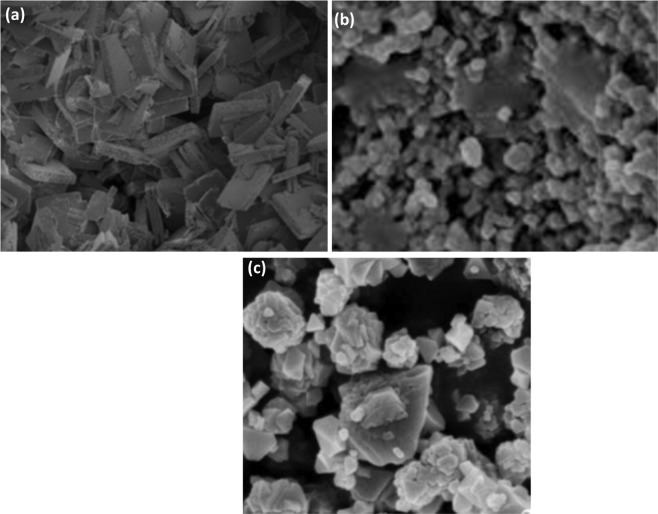
SEM images for: (**a**) NbOFFIVE-1-Ni, (**b**) SBA-15, (**c**) SIFSIX-3-Ni.

SEM micrographs for the three prepared materials (Fig. 3a–c) reveled clearly that the powders consisted of multi-sized agglomerations that ranges from 100–600 nm size for SBA-15, NbOFFIVE-1-Ni and SIFSIX-3-Ni. Smooth and uniform particle shapes were observed with voids present between granules. Ullah^3^ reported that in the case of SBA-15, tiny micrometer sized particles were present that might exist due to the high temperature used in the preparation and nucleation process. Although not tested in this study, Zhang^25^ conducted transmission electron microspores (TEM) and reported that the advanced testing clearly showed a two dimensional hexagonal opened framed structure confirming the low angle XRD results similar to the ones observed in our study (Fig. 3a) which may attribute to good CO_2_ sorption from gas streams.

### FTIR

**Figure 4:**
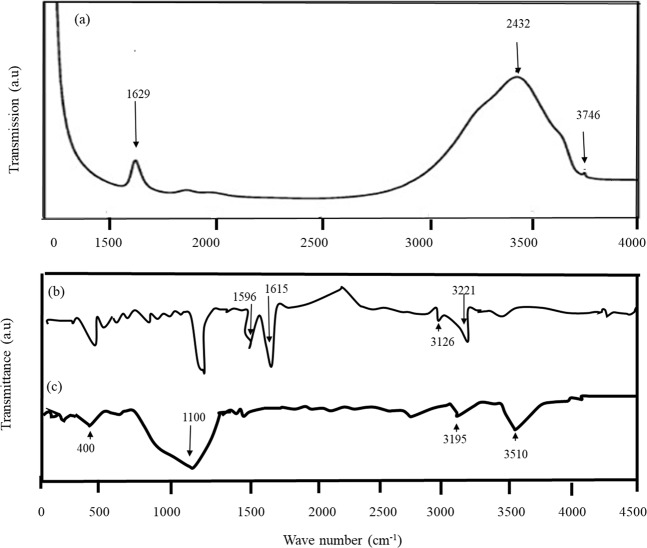
FTIR Spectrophotometry for (**a**) SBA-15, (**b**) NbOFFIVE-1-Ni, (**c**) SIFSIX -3-Ni.

FTIR spectroscopy assists in understanding the interactions between different species within the chemical composition of the materials. The FTIR spectra are recorded in the range 400–4000 cm^−1^. Figure 4 shows the FTIR spectrum of the prepared sorbents The FTIR of the SBA-15 shows the peak of silicate materials. The Si-O-Si bending, asymmetric stretching mode of SiO_4_ and Si-O stretching corresponds with the bands at 898 cm^−1^, 1110 cm^−1^ and 447 cm^−1^. Other peaks exist but at a much smaller level that are typically associated with the O-H stretching at around 3450 cm^−1^. This confirms the structure and purity of the prepared SBA-15 even when the preparation of the materials were altered by using microwave heating. The spectrum at around 3450–3500 cm^−1^ responsible to the stretching and vibration of intermolecular hydrogen bond (O-H). This is attributed to the presence of Si-OH groups present. The peak at 947 cm^−1^ is also due to the stretching vibration of Si-OH^26^. For NbOFFIVE-1-NI, peaks at 3221, 1615, 1402, and 465 cm^−1^ most likely correspond to the characteristic nickel O-H stretching.

#### Thermogravimetric analysis (TGA)

**Figure 5:**
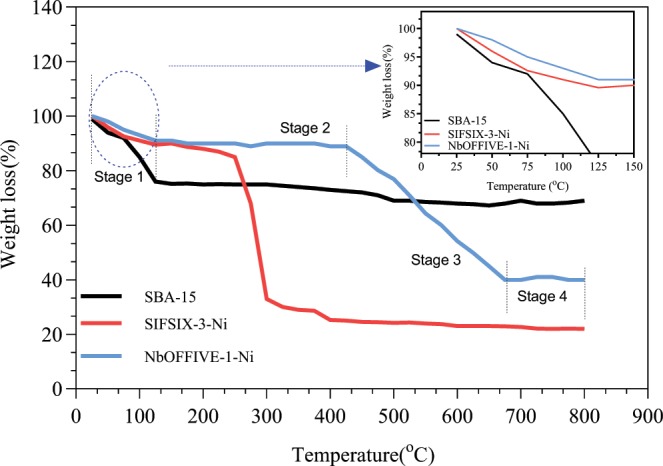
TGA analysis for three sorbents at a temperature range up to 800 °C.

The chemical, thermal and framework stability are essential elements in the selection of the appropriate HUM and mesoporous materials for separation applications. For CO_2_ adsorptive separations stability of the framework toward removing guest molecules from its pores is an essential^28^. In the context of this paper, thermal stability is considered here given the non-reactive nature between the CO_2_ and the sorbent as it is a limiting factor in hybrid framework materials and HUMs. To evaluate the thermal stability of the sorbents thermogravimetric analysis were conducted and in the temperature range between ambient and 700 °C. Plots of the change in mass of the sample at different temperatures are shown in Fig. 5. Steep slope is indicative of large mass loss and less thermal stability in the sorbent and could lead to material degradation. It can be seen from Fig. 5 that the three sorbents suffered weight loss at elevated temperatures. In addition, it seems that the weight loss trend occurred in a multistage manner that can be divided into three main areas: initial weight loss area, a plateau, a steep weigh loss area at elevated temperature. Although the trend is less obvious for the SBA-15, it holds true to the NbOFFIVE-1-Ni and SIFSIX-3-Ni compounds, each at a different temperature range.

Of the three samples, SBA-15 showed the most stability. The initial weight change occurred between ambient and 50 °C, before the mass change slope flattens. At around 400 °C a steep change is mass takes place before stabilizing around 500 °C. For NbOFFIVE-1-Ni, the first change in mass happened quickly between ambient and 100 °C. The weight loss stabilizes between 100–400 °C before changing steeply beyond the 400 °C but is lower than the trends observed for SBA-15. Bhatt^29^ reported on the preparation and fine tuning of fluorinated HUMs including NbOFFIVE-1-Ni. A 4-stage weight loss was observed with the first occurring between ambient and around 80 °C. In this period, less than 5% loss was perceived. The samples were then stable between 90–300 °C before disintegration and weigh loss occurred rabidly between 300–400 °C. The results of TGA obtained in our study were in good agreement of those reported in Bhatt^29^ and Kumar^22^. In the case of SIFSIX-3-Ni, steep degradation and mass loss occurred at a lower temperature compared to the two other sorbents namely at 245 °C. At the initial stage of weight loss, excess water or any other volatile components tend to evaporate. After this temperature, decomposition of the residual template on the wall surface can take place. Kumar^22,28^ reported similar trends for SIFSIX-3-Ni and showed that the samples were stable around a temperature of 250 °C after 10% initial weight change.

Although the trends of mass loss are reported in literature, the values at which the stages took place and decomposition occurs varied between reported studies. Zhang^25^ studies the effect of SBA-15 and its amine modified version in trace CO_2_ removal applications. In their work, SBA-15 did not show any significant mass loss in the temperature range between 25–600 °C and great thermal stability was observed. The amine-modified samples showed a typical three stage decline in mass and low thermal stability when temperatures exceeded 100 °C. In another study conducted by Ullah^3^ and co-workers, thermogravimetric analysis on commercial SBA-15 and amine modified versions showed a steep change simultaneous change in their thermal stability. The weight loss beyond 100 °C was less steep for both materials. The loss is attributed to the decomposition of surfactants and other impurities that may be available in the structure. Accordingly, it may be inferred that changes in the preparation procedure and the preparation methods and materials may affect the thermal stability of the material as no standard weigh loss has been reported in literature.

### N_2_ adsorption-desorption isotherms

**Figure 6:**
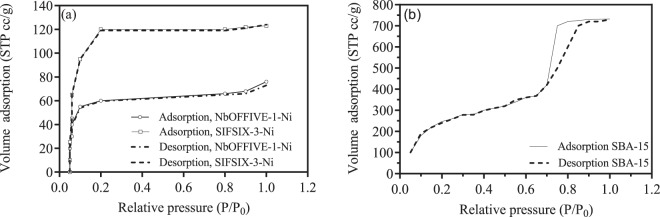
(**a**) N_2_ Adsorption (solid lines) and desorption (dashed lines) isotherms for NbOFFIVE-1-Ni and SIFSIX-3-Ni; (**b**) N_2_ Adsorption (solid lines) and desorption (dashed lines) isotherms for SBA-15.

The low pressure N_2_ adsorption-desorption isotherms obtained at 77 K are typically used to provide information on the associated microporosity-mesoporosity of the materials. The representative nitrogen adsorption–desorption isotherms for the three materials are shown in Fig. 6(a,b). The associated BET surface area, total pore volume and pore diameter are reported in Table 1 (as obtained from t-plots, not shown). Such properties are significant in the understanding of the interactions and separation applications of such materials amongst other properties such as the chemical structure. Accordingly, a large number of studies reported on the control of the porous structure of such sorbents via different synthesis routes, physical and chemical alterations of the structure^21,22,24,30,31^.

**Table 1:** Characteristics of sorbents.

Sorbent	BET (m^2^/g)	Pore size (nm)	Pore volume (cm^3^/g)	Type
NbOFFIVE-1-Ni	248	0.139	0.095	Crystalline microporous
SIFSIX-3-Ni	368	0.36	0.167	Crystalline microporous
SBA-15	527	6.6	9.31	Mesoporous silica

Figure 6(a) shows indicated a type I IUPAC classification indicating a dominant microporous structure^32^ compared to a classical IV shaped isotherm for the SBA-15 sorbent. The type I isotherm that was observed for both, indicate diffusion into the material structure by the sorbate is predominant. For NbOFFIVE-1-Ni and SIFSIX-1-Ni with no obvious hysteresis loop is shown (Fig. 6a) indicative of a lack of a mesoporous structure and contained mainly micropores. The typical IV shape in consistent with the hexagonal mesoporous structure of SBA-15 with one-dimensional cylindrical channels^33^. As indicated in Table 1, the surface area of the SBA-15 was the largest (527 m^2^/g) compared to 248 m^2^/g for NbOFFIVE-1-Ni and 368 m^2^/g for SIFSIX-3-Ni. The NbOFFIVE-1-Ni and SIFSIX-3-Ni are characterized with small pores and low porosity which offers them the required thermal stability as indicated in the TGA plots above.

Rashid^26^ reported that uniform hexagonal pores are characteristic of SBA-15 defined by a narrow pore size distribution and pore diameter of 5–15 nm giving the SBA-15 the required thermal and mechanical stability. The resultant BET surface obtained here fitted well with in the range of BET areas reported in literature for the highly ordered large-pore mesoporous silica molecular sieve SBA-15 (between 400–900 m^2^). The large variation in the surface area of the SBA-15 might be attributed greatly to the preparation methodology, raw material ratios and calcination temperatures. Having said that, the BET surface are of this material is still higher than many mesoporous materials belonging to the same class of materials^7,26^. The classical type IV isotherm (Fig. 6b) SBA-15 shows that the initial uptake occurs at the lower relative pressure (P/P_o_) less than 0.25. It can also be observed that a marked tall and narrow H1-shaped hysteresis effect (difference between the adsorption-desorption) occurs around the relative pressure range between 0.55–0.95 indicated of a well-structured mesoporous and capillary condensation structure^34^. The closure of the loop is thought to be related to the tensile strength effect of the material as reported by Beilstein^34^, where pores filled with N_2_ are drained with in the interconnected pores to smaller pores or sections of the material.

#### High-pressure CO_2_ adsorption

**Figure 7:**
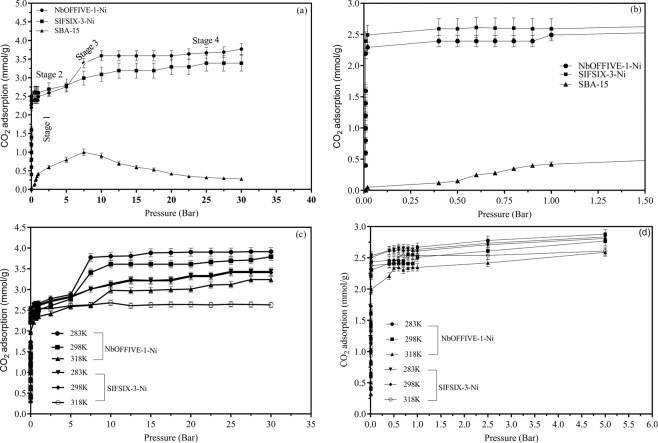
(**a**) High-pressure (0–35 bar) CO_2_ adsorption for NbOFFIVE-1-Ni, SIFSIX-3-Ni and SBA-15 at 298 K; (**b**) enlarged section of 7 (**a**) for pressures up to 1.5 bar; (**c**) High-pressure (0–35 bar) CO_2_ adsorption for NbOFFIVE-1-Ni, SIFSIX-3-Ni at different temperatures; (**d**) enlarged section of (**b**) for pressures up to 5 bar.

The experimental data of the CO_2_ excess adsorption capacity on the three selected sorbents are given in Fig. 7 at a pressure range from 1–30 bar and at three different temperatures (283, 298, 318 K), further elaboration on excess vs absolute adsorption amounts is given elsewhere^23^.

A general trend indicated that there is a marked difference between the CO_2_ uptakes of micro compared to meso-based sorbent materials. As indicated from the pore structure and pore volume results, the NbOFFIVE-1-Ni and the SIFSIX-3-Ni are mostly formed of micropores and there is a complete absence in the meso-structure. SBA-15 of the other hand is predominantly meso-porous and its analysis indicated no microposocity. The structure, micro and mesporocity, selection of the linkers and associated shape of the sorbent molecules can affect greatly the sorption capacity of the material^35^.

It can be observed that a sharp uptake of CO_2_ occurs for the three sorbents at the initial stages of adsorption (between 0–0.5 bar as indicated in Fig. 7b). The trends are classical in shape and shows a typical Langmuir shaped isotherm with immediate initial CO_2_ adsorption into the available sites in all three materials at all temperature ranges. The low-pressure initial sharp CO_2_ uptake corresponds well for the available adsorption cites for the three adsorbents. At a pressure between 2–7 bar, a marked step-change in the adsorption is noted for the three adsorbents. For the micropores based HUMs (Nb and SIF), the CO_2_ uptake shows continuous increase from 7 bar to around 20 bar beyond which the isotherm flattens and reaches saturation. For those two sorbents, four clear stages of adsorption are recorded: fast uptake between 0–0.5 bars followed by a plateau between 0.5–1 bars; a steep increase in CO_2_ uptake between 1–7 bar followed by another lower slope increase between 7–20 bar, and finally a saturation between 20–30 bar. The marked adsorption isotherm stages were more apparent in NbOFFIVE-1-Ni compared to that of SIFSIX-3-Ni. In the SIFSIX-3-Ni case, only three stages were observed: a typical Langmuir type adsorption isotherm between 0–1 bar, followed by a marked increase in CO_2_ capture capacity from 3–10 bar followed by another adsorption stage between 10–30 bar. When the BET surface areas are compared between the three sorbents, the adsorption capacity of SIFISX-3-Ni and NbOFFIVE-1-Ni are outstanding in comparison to SBA-15, where the reported BET surface area is 527 m^2^/g higher than the previous two. Although the expected trend related the high surface area with higher gas uptake capacity, it has been reported that no established (linear or non-linear) function exists to relate the surface area with uptake as a standalone function at a given operating pressure^36^. Here, the pore size and its distribution were reported to be more dominant in determining the type and behavior of the sorption isotherm for gases such as CO_2_^37^. This concept is applicable to the SIFISX-3-Ni and NbOFFIVE-1-Ni sorbents. While the SBA-15 showed a higher BET area, it did not pose a higher CO_2_ adsorption at pressures above 9 bar.

The sudden increase in the NbOFFI-1-Ni adsorption between 5–7 bar pressures (Fig. 7a,c) may be explained, in part, by what is referred to as gate opening adsorption^38^. The phenomena is related to the expansion of the cage structure with the presence of enough adsorbed gas up to a certain pressure where the pressure does not allow any further expansion. The type I Langmuir isotherm shown for SIFISX-3-Ni and NbOFFIVE-1-Ni favors a physiosorption rather than chemisorption behavior without any apparent gate-opening behavior at low pressure (up to 1 bar) rather than capillary condensation and increased density of CO_2_ beyond its critical pressure. The cross-linked cube structure may be responsible for strengthening the material at high pressure. In addition, pore volume may increase due to “swelling” under high pressure allowing CO_2_ to be introduced in the structure. When all available space is occupied, material saturation may occur and the levelling off plateau is observed for both NbOFFIVE-1-Ni and SIFSIX-3-Ni^38^.

Of the three sorbents, SIFSIX-3-Ni had the best performance under the high-pressure adsorption conditions at the same experimental temperature followed by NiOFFIVE-1-Ni. SBA-15 had a higher initial adsorption at low pressure. The ability to adsorb a meaningful amount of CO_2_ at higher pressures for SIFISX-3-Ni and NbOFFIVE-1-Ni indicate potential suitability to use such in applications related to CO_2_ capture from pre-combustion applications and even might be useful for temporary gas storage applications^39^. High selectivity towards CO_2_ is important to affirm further the suitability of the materials for pre-combustion applications. The selectivity of various gases are not included in the context of this paper. Additionally, Chen^40^ reported that are highly selective towards CO_2_ due to the suitable CO_2_ molecular fit in their ultramicropores (less than 0.7 nm), polarization in addition to electrostatic forces from their inorganic anions such as SiF_6_^−2^. The two are known to have a one-dimensional pore channels and adopt cubic topology.

**Figure 8:**
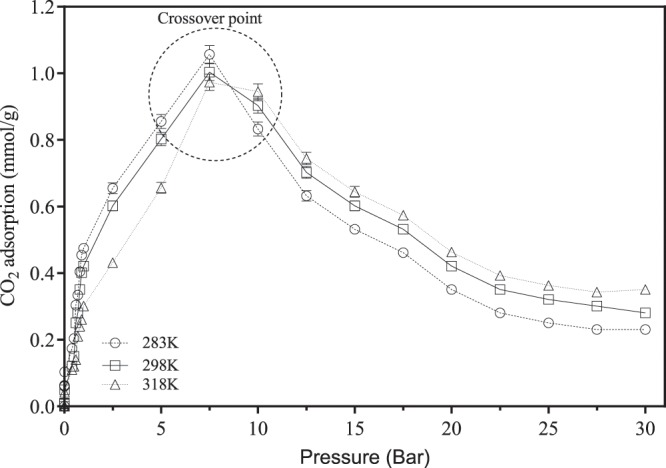
CO_2_ adsorption on SBA-15 at pressure range 0–35 bar and three different temperatures.

Interestingly, the overall adsorption isotherm trend observed for SBA-15 showed a completely different behavior than the other two sorbents under the same temperature and pressure conditions apart from the low-pressure region (0–1 bar). The isotherms (for the three temperatures) increased with the elevation of the pressure up to a maximum attained between 7–9 bar, depending on the temperature as shown in Fig. 8. At the inflection point (cross over region as shown in Fig. 8), a downward decrease in adsorption was shown between 8–9 bar. The CO_2_ uptake in mmol/g continues to decrease with the increasing temperature. It is noteworthy to mention that the increase in CO_2_ adsorption at the initial phase (between 0–max at 8–9 bar) showed clearly that the adsorption increased with the decrease in temperature up to the maximum value. Beyond this point, a switch in the adsorption behavior was prominent with higher temperatures resulting in higher CO_2_ adsorption (for the same pressure). A shift from the behavior is obtained under the same conditions at the lower pressure- temperature trend. This ‘crossover’ phenomenon was perceived for the SBA-15 and was not observed for either NbOFFIVE-1-Ni or SIFSIX-3-Ni where the lower temperatures were preferable for CO_2_ sorption at higher pressures.

The relationships between the temperature and the adsorption capacity trends at low pressure (for the three adsorbents) and at high pressure for the NbOFFIVE-1-Ni and SIFSIX-3-Ni are attributed to the exothermic nature of the adsorption process. At higher temperatures more energy is released via the adsorption process with negative impact on the on the strength of the binding forces between the materials and the CO_2_^15^.

The relationships and trends between temperatures and pressures for trends for either NbOFFIVE-1-Ni or SIFSIX-3-Ni have been reported in literature only for low pressures not exceeding 1 bar and in trace CO_2_ conditions (up to 400 ppm). Within the low range pressure alterations, the behavior and capacity of CO_2_ found in this study were within reported values given by^34,35,41,42^. To the best of our knowledge, no data were reported for the high-pressure adsorption of CO_2_ by NbOFFIVE-1-Ni and SIFSIX-3-Ni. Accordingly, the trends obtained cannot be ascertained or compared with other published data within the temperature and pressure ranges employed in this study. Having said that, Lee^15^ conducted a study on the CO_2_-CH_4_ selective adsorption on covalent organic polymers at high pressures using Rubotherm magnetic suspension microbalance at three different temperatures (298, 318, and 328 K) and pressures up to 55 bar. The trends showed a linear relationship between the pressure and the CO_2_ adsorption capacity. The authors did not observe any stepwise adsorption and the shapes of the isotherms were different from those reported for the like of HKUST-1, activated carbons and Na-zeolites^15^. Having said that the trends showed that the capacity for CO_2_ increased with the increasing in pressure while the temperature increase had a negative effect on adsorption (Figs. 7 and 8). The uptake amounts for CO_2_ using the reported organic polymers went beyond 3 mmol CO_2_/g and none of the isotherms indicated that no equilibrium was attained at 55 bar with an increase upward trend of uptake at higher pressures.

### Heats of adsorption

An important parameter in adsorption kinetics is the isosteric heat of adsorption (Q_st_); a value that indicates the quantity of the heat released in sorbent-sorbate interaction and is a measure for the intensity of such interaction. Positive values indicate endothermic interactions while higher numerical values indicate stronger interactions between CO_2_ and the sorbent material. Smaller heats of adsorption requirements are preferable as they indicate the ease of regeneration of the sorbent for proper use in industrial adsorption processes. Highly selective physisorption advanced sorbents for CO_2_ capture offer great ability as they require lower energy for recycling^42^.

**Figure 9:**
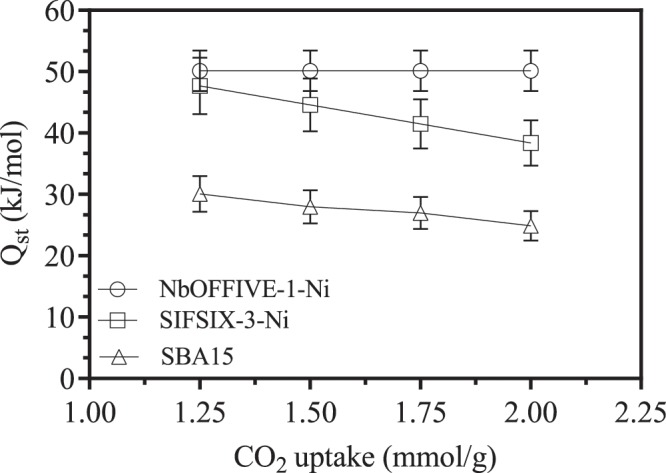
Isosteric heats of sorption for CO_2_ on NbOFFIVE-1-Ni, SIFSIX-3-Ni and SBA-15.

Isosteric heats of sorption-based on Clausius-Calapeyron equation were calculated from variable temperature adsorption isotherms based on linear plots obtained from ln P vs 1/T^23^. For NbOFFIVE-1-Ni (Fig. 9), Q_st_ decreased in value initially as the loading amounts increase. The similar initial trend is shown in^22^. Typically, a high value of Q_st_ for a single gas adsorption is indicative of excellent performance for the gas capture (CO_2_ in this case). A value of less than 80 kJ/mol indicate strong domination of physisorption rather than chemisorption. In such cases, regeneration is easier and requires less energy input favoring the use of such materials for CO_2_ capture. The dominance of the physisorption is in line with results obtained by N_2_ adsorption isotherms. Bhatt^26^ reported that NbOFFIVE-1-Ni offers superior CO_2_ volumetric uptake using physisorption mechanisms at low CO_2_ concentrations. The initial low values in Q_st_ could be attributed to strong CO_2_ adsorption at the start of the process when the NbOFFIVE-1-Ni cube structure is empty and have free space to accommodate CO_2_ within its cage structure. In addition, the slight initial decrease in Q_st_ with amount absorbed for NbOFFIVE-1-Ni between 0.1–0.8 mmol/g has been replaced with respect to some energetic surface heterogeneity Lee^15^. Bhatt^29^ related the strong physisorption and affinity for CO_2_ can be explained via analyzing the structure-property relationship between NbOFFIVE-1-Ni and CO_2_. SCXRD and interpretation of the Fourier difference data indicated a localization effect in the CO_2_ molecules in the NbOFFIVE-1-Ni’s square-arranged channels. In the SIFSIX-3-Ni, a slight increase in the heat of adsorption with the amount absorbed might be explained by the intermolecular attraction forces^15^.

The physisorption is stronger in NbOFFIVE-1-Ni than that in case of SIFSIX-3-Ni possibly due to weaker SIF-CO_2_ framework interactions. Such analysis places emphasis on the importance of tunable structures in favor of CO_2_ capture and storage applications. For SAB-15, the Q_st_ value was around 20 KJ/mol. The results are consistent with reported values obtained by Reiser^23^. A slight decrease in the Q_st_ is noted before steadying over the range of adsorbed moles. Similar behavior was reported by Ullah^3^. The unoccupied, freely available initial cites in the SBA-15 have a stronger affinity to the CO_2_ molecules within the surface and the pores of the material.

## Conclusions

HUMs highly crystalline structure and ability to be fine-tuned and pore-size controlled have a superior ability to adsorb CO_2_ under high and low pressures. To our knowledge, first-time high-pressure values were reported up to 35 bars for NbOFFIVE-1-Ni and SIFSIX-3-Ni. Multistage adsorption was reported especially with the NbOFFIVE-1-Ni. At 1 bar, the CO_2_ adsorption abilities were in the order SIFSIX-3-Ni (2.6 mmol/g)>NbOFFIVE-1-Ni (2.4 mmol/g) >SBA-15 (0.41 mmol/g). Micro-mesostructure rather than surface area place a pivotal role in the enhanced capacity together with the electrostatic affinity offered by the two HUMs. At pressures higher than 1 bar, a multi-stage absorption was observed with initial step CO_2_ uptake followed by slower adsorption. Increasing the temperature at the range of pressure (0–35) reduced the CO_2_ uptake in both HUMs indicative of the exothermic nature of the adsorption. SBA-15 temperature effect was similar in pressures up to values between 5–7 bars after which a cross over effect is observed and temperature dependence is reversed (i.e., higher temperatures resulted in higher adsorption in this mesoporous material).
